# Supplementary material for: Family-Assisted Severity of Illness Monitoring for Hospitalized Children in Low-Resource Settings—A Two-Arm Interventional Feasibility Study
Source: Front Pediatr. 2022 May 23;10:804346. doi: 10.3389/fped.2022.804346 (PMC9169086; doi:10.3389/fped.2022.804346)
Supplement: Supplementary file 1 [file Table_1.docx]

**Supplemental Table 1**: Number of recorded clinician assessments per hour in intervention vs control group over the first 24hr enrollment period from 06:00 to 22:00

| Recorded Number of  clinician visits at patients’  bedside per hour | Control  n (%) | Intervention  n (%) |
| --- | --- | --- |
| 0 | 0 (0) | 375 (33) |
| 1 | 311 (31) | 308 (27) |
| 2 | 86 (9) | 134 (12) |
| 3 | 13 (1) | 16 (1) |
| 4 | 5 (1) | 3 (0) |
| 5 | 1 (0) | 0 (0) |
| 7 | 0 (0) | 1 (0) |
| missing | 589 (59) | 298 (26) |
