# Supplementary material for: Family-Assisted Severity of Illness Monitoring for Hospitalized Children in Low-Resource Settings—A Two-Arm Interventional Feasibility Study
Source: Front Pediatr. 2022 May 23;10:804346. doi: 10.3389/fped.2022.804346 (PMC9169086; doi:10.3389/fped.2022.804346)
Supplement: Supplementary file 2 [file Table_2.DOCX]

**Supplemental Material**: Exit surveys for FASTER Clinicians (A) and Caregivers (B)

| **PTID**  **(A) Exit Survey – FASTER medical providers Form completion date:______________________** | |
| --- | --- |
|  | What is your role?   - Doctor - Medical officer - Clinical officer - Nurse - Nursing student - Other: _______________ |
|  | How many work days did you spend with parents involved in assisting in patient monitoring?   - 1-5 days - 6-10 days - 11-20 days - More than 20 days |
| **3.** | How was your experience with the parental monitoring tool in general? |
| **4.** | Did the parents capture their child’s severity of illness and respond adequately? |
| **5.** | What were the main challenges of this intervention from your perspective?  Increased workload  False signaling  Parents more demanding  Other, please specify: __________________________________________ |
| **6.** | How could we improve this intervention?  _______________________________________________________________________ |
| **7.** | Do you think this intervention is challenging  for the parents and child? Yes No  **7a.** If yes, please explain in what way:  ____________________________________________________________________ |
| **8.** | Do you think this intervention would improve care of a very sick child in resource-limited settings?  Yes No Don’t know  **8a.** If yes, please explain your response.  ____________________________________________________________________ |

| **PTID**  **(B) Exit Survey/Interview for Caregivers** | |
| --- | --- |
| **1.** | Date of exit survey:      Completed as Survey Interview ***DD*** ***MM*** ***YR*** |
| **2.** | Gender:  M F |
| **3.** | How was your experience with monitoring your child in this study? |
| **4.** | 4a. Was the training easy to understand for you?  Yes No Don’t know  4b. If no, what was difficult and how could it be improved? |
| **5.** | Did the doctors and nurses respond as you would  have expected to the flag signs? Yes No  If no, what would you have wanted them to do differently: ______________________ |
| **6.** | How did the doctors and nurses treat you during the monitoring process? |
| **7.** | What were the main challenges of monitoring your child?  none  Discomfort  Fatigue  Stress  Interaction with doctors or nurses  Other________________________________________________________ |
| **8.** | How could we improve this intervention? |
| **9.** | Do you think caregiver monitoring would improve  care of a very sick child in this setting? Yes No Don’t know  ***9a***. If yes please explain your answer. |
| **11.** | Do you think the monitoring skills you have learned will help you recognize a sick child in your community?  Yes No Don’t know |
