# Supplementary material for: Family-Assisted Severity of Illness Monitoring for Hospitalized Children in Low-Resource Settings—A Two-Arm Interventional Feasibility Study
Source: Front Pediatr. 2022 May 23;10:804346. doi: 10.3389/fped.2022.804346 (PMC9169086; doi:10.3389/fped.2022.804346)
Supplement: Supplementary file 3 [file Table_3.DOCX]

**Supplemental Table**: Number of clinician assessments per hour in intervention vs control group over the first 24hr enrollment period. Blank hourly caregiver data forms were counted as no clinician visits.

| Number of nurse and physician visits at patient’s bedside per hour | Control  n (%) | Intervention  n (%) |
| --- | --- | --- |
| 0 | 980 (65) | 1148 (67) |
| 1 | 384 (26) | 380 (22) |
| 2 | 116 (8) | 151 (9) |
| 3 | 15 (1) | 18 (1) |
| 4 | 5 (0) | 4 (0) |
| 5 | 1 (0) | 0 (0) |
| 7 | 0 (0) | 1 (0) |
| Number of physician visits at patient’s bedside per hour | Control  n (%) | Intervention  n (%) |
| 0 | 1246 (83) | 1406 (83) |
| 1 | 238 (16) | 276 (16) |
| 2 | 15 (1) | 19 (1) |
| 3 | 2 (0) | 1 (0) |
| Number of nurse visits at patient’s bedside per hour | Control  n (%) | Intervention  n (%) |
| 0 | 1118 (74) | 1284 (75) |
| 1 | 358 (24) | 399 (23) |
| 2 | 21 (1) | 16 (1) |
| 3 | 4 (0) | 2 (0) |
| 5 | 0 (0) | 1 (0) |
