# Supplementary material for: Family-Assisted Severity of Illness Monitoring for Hospitalized Children in Low-Resource Settings—A Two-Arm Interventional Feasibility Study
Source: Front Pediatr. 2022 May 23;10:804346. doi: 10.3389/fped.2022.804346 (PMC9169086; doi:10.3389/fped.2022.804346)
Supplement: Supplementary file 4 [file Table_4.DOCX]

**Supplemental Table**: Univariate Effects for main model

| **Effect** | **Relative Change (%)** | **95% Confidence** | **P-value** |
| --- | --- | --- | --- |
| Female vs. Male | -2.7 | (-22.6,22.3) | 0.812 |
| Admission PEWS (per point) | 5.9 | (2.9,8.9) | <0.001 |
| Intervention vs. Control | 5.1 | (-16.2,31.9) | 0.666 |
| Severity Effect (Red vs. other) | 49.3 | (19.9,86.0) | <0.001 |
| Age <6 months | 38.1 | (-2.5,95.6) | 0.069 |
|  |  |  |  |
| Parent Education |  |  |  |
| Primary vs. Higher Level | -17.0 | (-34.8,5.7) | 0.131 |
