# Supplementary material for: Family-Assisted Severity of Illness Monitoring for Hospitalized Children in Low-Resource Settings—A Two-Arm Interventional Feasibility Study
Source: Front Pediatr. 2022 May 23;10:804346. doi: 10.3389/fped.2022.804346 (PMC9169086; doi:10.3389/fped.2022.804346)
Supplement: Supplementary file 5 [file Table_5.DOCX]

**Supplemental Table**: Patient Mortality by covariates

| **Covariate**  **Sex** | **Survived**  **n (%)** | **Died**  **n (%)** | **Total**  **n (%)** | **P-value** |
| --- | --- | --- | --- | --- |
| Male | 71 (91) | 7 (9) | 78 (100) | 0.43 |
| Female | 52 (85) | 9 (15) | 61 (100) |  |
| Sum | 123 (88) | 16 (12) | 139 (100) |  |
| **Language** |  |  |  |  |
| Swahili | 76 (90) | 8 (10) | 84 (100) | 0.53 |
| English | 47 (85) | 8 (15) | 55 (100) |  |
| **Education** |  |  |  |  |
| Primary | 40 (85) | 7 (15) | 47 (100) | 0.57 |
| Secondary | 60 (90) | 7 (10) | 67 (100) |  |
| Certificate | 9 (82) | 2 (18) | 11 (100) |  |
| Diploma | 13 (100) | 0 (0) | 13 (100) |  |
| Degree | 1 (100) | 0 (0) | 1 (100) |  |
| Sum | 123 (88) | 16 (12) | 139 (100) |  |
